# One Way to Fame; or Dr. Morton and His “Letheon”

**Published:** 1895-02

**Authors:** 


### ONE WAY TO FAME; OR DR. MORTON AND HIS "LETHEON."

We are in receipt of some "Memoranda Relating to the Discovery of Anesthesia," from which we learn that the name of Dr. W. T. G. Morton, "the discoverer of the safe use of ether," will be "enrolled upon the base of the dome in the new chamber of the House of Representatives in the State House in Boston, among the selected fifty-three of Massachusetts' most famous citizens," and we are requested by friends of Dr. Morton to make such notice of this new honor to his memory as we may think proper. We cheerfully do so.

There are ways and ways to fame. Dr. Morton's claim to greatness seems to have proceeded from the following facts: That while a medical student in the office of Dr. Chas. T. Jackson, he received from the latter gentleman a suggestion that the vapor of sulphuric ether might be used to prevent pain in the extraction of teeth; that he acted upon and verified the suggestion; that within twenty-four hours he consulted a lawyer (Mr. Eddy) in order to obtain letters patent upon the "new gas"; that Mr. Eddy concluded that Dr. Jackson was properly entitled to the credit; that Dr. Jackson would not allow his name to be associated with the patent, but resigned his interest to Morton for ten per cent. of all the latter could make out of it; that a patent was secured both in this country and England; that the "new gas" was tested with success in the Massachusetts General Hospital, October, 1846, and called "letheon"; that Morton tried to disguise the vapor of the ether by the addition of aromatic oils, etc.; that after two or three operations in the hospital the surgeons declined further use of the gas until informed by Morton what it consisted of; that Morton admitted it to be only sulphuric ether; that he proceeded (through his lawyer) to sell it to individuals for twenty-five dollars per quart, and the privilege of using it for five years for one hundred dollars; that he made several unsuccessful appeals to Congress for remuneration; that in a short while the government even nullified the letters patent, etc.

Naturally enough the uses which Morton proposed to make of his discovery were far from being indorsed by the medical profession. A writer in the Boston *Medical and Surgical Journal* (December, 1846) said: "It seems unfortunate, to say the least, that the discovery has not been brought to public notice in a different manner, and under different circumstances. We are told that it is *patented*. What is patented? The operation of a well-known medical agent? I doubt the validity of such letters patent. . . . It should be free to all who can do good with it, or receive relief from suffering by it. To patent it would be what it would have been for Jenner to have patented vaccination."

Dr. Morton's course is approved no more to-day than it was then. Truth and right are the same for all time. It is said that only one medical journal in the country supports the claim of Dr. Morton as the original discoverer of anesthesia. On Boston Common there is a handsome monument erected by a friend of Dr. Morton, "To the Discoverer of Anesthesia," but Boston declined to put Morton's name on it, and when the monument was unveiled a few years ago the mayor of the city, in accepting it, never once alluded to Dr. Morton. It stands there unnamed to-day; a monument, not to a man but to a fight—an inglorious fight for glory, and a pauperizing scramble for money.

Dr. Long and Dr. Wells made original discoveries, unassisted by anybody else, and to them belongs the credit which for years has been undeservedly bestowed upon Dr. Morton. But medical history is being adjusted now by impartial writers, and all the claimants are going down to history in their true relations with this matter. On this subject the writer received some months ago a letter from a well known physician in Boston, which closed thus: "Too much credit has been given to the dashing empiric instead of to meritorious and modest investigation."

---

THE Rome Society of Medicine has elected the following officers for the ensuing year: Dr. L. P. Hammond, President; Dr. R. B. Harbin, First Vice-President; Dr. J. C. Garlington, Second Vice-President; Dr. Frank Wynn, Secretary; Dr. C. Hamilton, Treasurer. The society is made up of Floyd county physicians, and meets twice monthly.

We would be pleased to hear from this society in our pages.

